# Mind, Matter, and Medicine

**Published:** 1892-11-12

**Authors:** 


### The Doctor's Armchair.

### MIND, MATTER, AND MEDICINE.

Where does matter end and mind begin? And what has medicine to do with mind? These are the sort of questions that philosophy loves to ask. For the present we may be content with a less ambitious consideration of the subject than philosophy would put us upon. If anybody could really tell us where and how mind is marked off from matter we would willingly be at the pains to learn all about it. But as nobody yet seems to have even come near finding out, and as the writer himself has no special call to metaphysical exercitation, it will be as well to avoid unnecessary waste of time. The other question, "What has medicine to do with mind?" hardly lends itself to philosophical discussion in a two-column article either. As questions of solid and sober fact, we all know that mind and matter have a very profound influence upon each other, and some of the most notable and worthy triumphs of modern medicine have been made in mental cases. Let us therefore discuss the subject practically and with common sense —honest common sense, more and more beloved and trusted as men and women grow in experience.

A good deal of the severest suffering of the present day is mental suffering. To many of the men and women, who are now doing the world's work and fighting the world's battles, the pains of the mind are exceedingly familiar. Yet they are not pains of the mind altogether either, but rather mental culminations of physical origin. To take one of the commonest of present-day forms of mental suffering—depression of spirits; this, in many cases, springs from purely physical roots, and not seldom its beginnings are so comparatively insignificent that a five-grain blue pill is sufficient to give them effectual check. But the depressions of spirit which are of a profound and persistent character can by no means be so easily disposed of. They constitute a real and serious evil—an evil which often paralyses business capacity, and destroys all the happiness of life. The body is not felt to be the impaired part of the man; it is the mind upon which the suffering is concentrated. If the victim could but throw off the load which is weighing him down; if he could but feel hopeful and confident as of yore; if he could but shut a door upon all his troubles, even when he goes to bed at night, and for once enjoy the careless, dreamless, deep, and delicious slumber of boyhood, he thinks he could be a happy and contented man.

Irritability is another acrid mental fruit which often springs from a material stem. It is not natural to the well developed and healthy man to give displays of irritable temper any more than it is natural for the strong young horse or lamb to lie down in a sulky fit instead of prancing and gambolling about in an exuberance of physiological delight. And yet, the wholesome-hearted boy, who was the joy of the cricketfield, sometimes becomes the soured and waspish man who is the nuisance and terror of the dinner table or the drawing room. When the high-spirited and joyous boy finds himself at forty-five a peevish, discontented and unjoyous man he has reasons for concluding that there is something seriously wrong with him. He probably blames his temper, or attributes the evil to his circumstances. If he were wise he would call his physiological creditors together and discuss affairs with them. Perhaps he has incurred a heavy debt to sleep, and now finds himself quite unable to repay it. He cannot sleep as many hours of the twenty-four as nature ought properly to demand, and so his brain suffers from excessive sensitiveness and abnormal reflexiveness. The brain of the middle-aged man is frequently roused to activity by subjects which demand most of all patient consideration. But the brain whose reflexiveness is too sensitive never can give patient consideration to a subject. It cannot help responding to stimulus by immediate action, and such action is of the purely impulsive kind, and is as often wrong as right. Indeed, it is entirely a matter of accident whether it be right or wrong. If the brain happens to be in a wholesome train of activity its reflex operations in response to stimuli will, as a rule, be wholesome; but if on the other hand the brain be working on lines of unwholesome activity, its reflexes will be unwholesome, and that without any regard whatever to the nature of the stimuli which excite them. But this is the beginning of insanity. When a really powerful brain is the creature of stimuli and reflexes, and has practically abandoned sober and patient thought, it has given itself up to a demon whose name in the long run is discovered to be "Madness." It may not be the kind of madness which requires control in a madhouse. But madness it is in its true essence, and it is fatal to further progress on the lines of wholesome and effective living.

Excessive brain activity is a creditor which demands payment in full in the long run, although he may at first be content with ten or twenty years' bills, and even renew them occasionally. But payment in coin, and payment in full, with interest, he insists upon, and extorts before the transaction is finally closed. The will fights many a battle with the capacity; but, though the will may give the capacity a thousand falls, it is the capacity which at the winding up gives the true measure of the victory or the defeat. No man can get more out of himself than Nature has put in. He who was designed in the physiological workshop for a gerund-grinder will never be a poet, and he whose capacity is no more than that of a superior lawyer's clerk need not expect ever to govern the State.

The man who takes the justest measure of his own bodily and mental capacity for work, and acts accordingly, is he who will bring his life wares finally to the best market. Darwin had a small capacity for work, but then he knew it, and never overworked either body or mind. He worked systematically, and strictly according to his measure, with the result that he produced ten times more literary work than the average writer, and of a hundred times better quality.

All this is the very A B C of modern medicine. Many people who admit it all in theory, when taken on their reasoning side, scout it absolutely in practice. Capable financiers are they in matters monetary, but the maddest of spendthrifts in the matter of health. The mind has to work through the body, and hence it is the subject of manifold limitations. The really intellectual man is his own physiological banker, and he manages his bodily and mental affairs so well that he not only avoids bankruptcy, but increases in various kinds of physiological wealth as he grows older. He who so manages the affairs of his body and mind as to become a physiological bankrupt before old age is generally as truly an incapable man as he who becomes bankrupt in finance.

